# The Medical Work of the Education Department

**Published:** 1915-03-27

**Authors:** 


### THE PUBLIC HEALTH OF LONDON.

### The Medical Work of the Education Department.

The new report of the public-health work of the London County Council (for the year 1913) fully maintains the reputation established by its predecessors. In the wealth of its statistical details, the fulness of its records, and its general interest it is far in advance of the similar reports of other English counties. The volume covers both the ordinary public-health work of the Council and the medical work of the Education Department, and it is interesting to note that the report of the latter occupies one and a half times as much space as that of the former. It is impossible, within the limits of our space, to do more than draw attention to a few of the more interesting items in these voluminous reports.

It will probably surprise many people to hear that the population of London is slowly decreasing. The Census population in 1901 was 4,536,267; in 1911 it had fallen to 4,521,685, and in 1913 the estimated population was 4,518,911. The report points out that the cause of this decrease is to be found in the outward movement of the population over the county boundaries. The result has been not only a diminution in numbers, but an alteration in the age and sex constitution of the population—an alteration much more marked in the districts north of the Thames than in those on the south, owing to the fact that the former area was earlier built over than the latter. A study of the statistics given in the report shows that this emigration from the county area has resulted in a disproportionate loss of the younger population and an increase of the female population between 25 and 40.

The marriage rate in 1913 fell slightly to 18.3 per 1,000, but with the exception of 1912, when the rate was 18.6, it is the highest since 1899. The birth rate was 24.5 per 1,000, as in 1912, and thus for the first time in ten years no decline is recorded. The death rate was 14.2, a slight increase on 1912, owing mainly to the fact that the infantile mortality rose from 91 to 105. A decrease occurred in the death rates of all the principal epidemic diseases. There was an increase in the number of cases of scarlet fever, diphtheria, and erysipelas notified, and a decrease in the case of smallpox, typhoid fever, and puerperal fever. Four cases of smallpox were reported, but in only one case was the diagnosis confirmed by subsequent observation. There was a large increase in the number of cases of scarlet fever, but the epidemic was of an extremely mild type. Attention is drawn in the report to an investigation made by Dr. Millson in Southwark, from which he concludes that if a child escapes scarlet fever up to the age of five years it stands a very fair chance of escaping altogether. Eight cases of typhus fever were notified—six of them from one house in Stepney. Details are given of these six cases, and it is pointed out that they probably all suffered from the same disease, but there was considerable divergence of view among medical men who saw them as to the diagnosis, and that it is even now doubtful whether the illness was typhus or typhoid fever.

The report on the medical inspection of school children during the year under review shows the great amount of work which is now being done under this head. Thirty full-time and six quarter-time medical men, working under the direction of four divisional medical officers, were employed. The number of children examined was 226,215, and of these more than one-third—viz., 86,787—needed treatment for various defects. In addition, a special examination of the teeth of 31,858 children was made by qualified dentists, and 25,492 were found to require dental treatment. During the year the facilities provided by the Council for treatment were increased, so that provision has now been made at twelve hospitals and twenty-two school centres for the treatment of 84,350 children. The establishment of further centres is contemplated, which will have the effect of increasing to an approximate total of 100,000 the number of children for whom facilities for treatment of one kind or another are provided. The rapidity of the growth of this department of the Council's work may be judged from the fact that at its inception in January 1910 the arrangements made were with six hospitals for 12,000 children. When the full scheme is working it will be possible for the Council to provide treatment for every child found during the medical inspection to require it. In 1913, 21,464 children received treatment for diseases of the eye, 13,618 for diseases of the ear, nose, and throat, 1,646 for ringworm, 20,130 for dental defects, and 10,159 nursing treatment. Comparing these figures with the provision made, it will probably be necessary to increase the facilities for ophthalmic, nursing, and ringworm treatment, whilst those for the treatment of diseases of the nose, ear, and throat would appear to be sufficient. In discussing the provision of medical treatment, the County Medical Officer points out that, whilst the general hospital is a most important element in the construction of a complete scheme, the medical-treatment centre possesses greater elasticity, and lends itself, in some respects, more readily than the hospital to the needs of the education authority. Hospital treatment does not allow of effective after-care, and particularly in minor ailments is of necessity unsatisfactory. In these cases daily treatment of a simple kind is required, and can be best carried out at a school clinic by a trained nurse under medical supervision. With the exception of a slight modification in the case of the dental centres, the Council decided to continue the financial arrangements made with the hospitals and treatment centres. These consist of payments made on the basis of £50 per annum for each doctor, surgeon, or anæsthetist employed on one half-day a week and *pro rata* for every half-day so worked, with an additional capitation payment of

2s. for each major ailment treated in respect of defects of the eye, ear, nose, and throat, and 7s. for the $x$-ray treatment of ringworm.

The report includes several interesting special investigations. Dr. Woods conducted an inquiry into the relation between nasal obstruction and protrusion of the upper incisor teeth. He examined 1,175 children, of whom eighty-eight showed marked protrusion; of these thirty-nine showed no evidence of adenoids. In forty-seven of the cases the mother was seen and examined. Of these forty-seven mothers, twenty-seven showed obvious protrusion of the teeth and twenty-four admitted using a "comforter" for the children. The general conclusions formed by Dr. Woods were that protrusion of the teeth is not always associated with adenoids, that the statement that there is always a history of the prolonged use of the "comforter" in cases not associated with adenoids is not strictly correct, and that heredity is an important predisposing factor.

Dr. McHattie investigated the frequency of occurrence of mirror writing in normal school children. The condition is most frequently associated with mental deficiency and with left-handedness. The left-hand writing of 1,240 children was examined, and only seven instances of mirror writing were discovered. In no case was there any reversal with the right hand. Interesting facsimiles of this mirror writing are included in the report.

Other interesting reports are Dr. Palgrave's, on faulty home management in relation to malnutrition; Dr. Norman's, on a comparison of the nutrition of children who had been breast- or bottle-fed as infants; and Dr. Mabel Russell's, on the conditions as to sleep of some Stepney school children.

